# The Iron Enigma: Expounding Iron Deficiency in a Pregnant Woman With Hemochromatosis and Celiac Disease

**DOI:** 10.7759/cureus.71926

**Published:** 2024-10-20

**Authors:** Zahid Idrees, Hashim Khan, Muhammad Zain Akhtar, Usman Khan

**Affiliations:** 1 Gastroenterology and Hepatology, Lancashire Teaching Hospital, Preston, GBR; 2 Internal Medicine, Lancashire Teaching Hospital, Preston, GBR; 3 Medicine, Royal Preston Hospital, Preston, GBR; 4 Internal Medicine, Royal Preston Hospital, Preston, GBR

**Keywords:** antenatal care, celiac disease, hereditary hemochromatosis (hh), iron deficiency anemia (ida), pregnancy

## Abstract

Hereditary hemochromatosis (HH) is an inherited disorder, characterized by abnormal iron metabolism, resulting in systemic iron overload. The iron deposits in multiple organs including the liver, pancreas, heart, pituitary gland, joint, and skin and causes tissue damages leading to clinical manifestations. On the contrary, celiac disease (CD) is an autoimmune disorder associated with genetic and environmental triggers and frequently associated with iron deficiency. Additionally, pregnancy results in a physiological state of increased iron requirement due to volume expansion and fetus growth and development. We present an interesting case of a young female patient with concomitant CD and HH, who developed iron depletion following venesection which was further complicated by pregnancy and the postpartum period.

This case highlights the rare co-existence of CD and HH, underscoring the diagnostic and therapeutic challenges in managing these concurrent conditions. Furthermore, it emphasizes the importance of patient education, clinical management, appropriate follow-up, and close monitoring of patients by multidisciplinary teams to establish the correct balance of body iron storage in order to prevent potential complications in a female patient of child-bearing age.

## Introduction

Hereditary hemochromatosis (HH) is one of the most common inherited disorders among the Northern European population and is characterized by genetic susceptibility to increased iron absorption with subsequent systemic iron overload and toxicity [1]. It is inherited in an autosomal recessive fashion and is commonly associated with mutations of HFE H63D and C282Y genes. Patients who inherit the abnormal HFE C282Y gene in a homozygous fashion are at an increased risk of developing iron overload due to decreased levels of hepcidin which results in the increased absorption of iron [2]. HH is a multisystemic disorder that is usually diagnosed in the 40s and 50s with women diagnosed later than men, likely due to blood loss from menstruation and pregnancy. A common presentation is fatigue with mildly deranged liver function tests. Serum ferritin and transferrin levels are the key in the diagnosis, and cutoffs vary by gender with ferritin levels of >300µg/L and transferrin saturation of >50% in men and >200µg/L and 40% in women considered highly suggestive of hemochromatosis and, in the absence of other causes, warranting genetic testing. The mainstay of treatment is venesection with a targeted ferritin level of <50µg/L and a transferrin saturation of <50%. While on induction treatment, the British Society of Gastroenterology advises weekly full blood counts (FBC) and monthly ferritin checks +/- transferrin. If diagnosed and treated early, it has no bearing on overall life expectancy [2,3].

On the contrary, celiac disease (CD) is an immune-mediated enteropathy triggered by gluten, a protein commonly present in wheat, barley, and rye, and frequently associated with iron deficiency anemia. Its development in patients is multifactorial with an increased propensity in people with human leukocyte antigen (HLA) DQ-2 and DQ-8 subtypes [4]. Iron deficiency in CD is multifactorial and may be due to villous atrophy or blood loss. Common presentations of CD include diarrhea, weight loss, and iron deficiency anemia. Serological tests such as anti-tissue transglutaminase (anti-TTG) and anti-endomysial antibodies are used for initial diagnosis with confirmation done via upper gastrointestinal (GI) endoscopy and biopsy which show partial or complete villous atrophy. The treatment revolves mainly around the elimination of gluten from the diet which is life-changing for many patients and can often lead to significant frustrations in patients [5,6].

Iron through its role in oxygen delivery and the electron transport chain has a critical role in the body. The demand for iron changes in pregnancy due to volume expansion in the mother and increased demand from the fetus and placenta as well as increased iron loss [6]. Gestational iron deficiency is associated with adverse pregnancy outcomes, including maternal illness, low birth weight, prematurity, and intrauterine growth restriction [7]. Therefore, iron monitoring and replenishment are required for favorable pregnancy outcomes.

The pathophysiology of iron absorption and storage is complex and mediated by multiple regulatory proteins. Iron absorption from the duodenum is mediated by the divalent metal transporter 1 (DMT1). In enteric mucosal epithelial cells, ferrous iron converts enzymatically to the ferric form and is transported via circulation to the macrophages and hepatocytes for metabolism and storage. The iron influx into cells is mediated by the transferrin receptor 1 (TfR1), while efflux is dependent on ferroportin. The HFE protein regulates hepcidin, the systemic iron regulatory hormone. Hepcidin peptide inhibits the export of iron from enterocyte, hepatocyte, and reticuloendothelial system (RES) macrophages by inducing the degradation of the iron-exporter ferroportin receptor. Hepcidin levels are suppressed in HH, leading to reduced ferroportin degradation and increased iron transfer into the circulation from these cells resulting in iron overload. For these reasons, it is quite uncommon for patients with HH to develop an iron-deficient state [8].

However, the co-existence of HH and CD in patients is rare and due to the conflicting pathophysiology can pose challenges and may lead to a presentation of an iron-deficient state as seen in a few case reports done in this small patient population. We report here another intriguing case of a 38-year-old Caucasian female who developed iron depletion after undergoing three sessions of venesection which was further complicated by pregnancy and lactation.

## Case presentation

A 38-year-old Caucasian female patient was reviewed in the gastroenterology follow-up clinic 10 months postpartum in view of iron deficiency anemia on the background of CD as well as HH. Her hemoglobin (Hb) was 128g/dL and her ferritin was 8µg/L on this visit (Table 1).

**Table 1:** Clinical parameters and normal values of biochemistry results. Hb: hemoglobin

Parameter	Pre-venesection	Post-venesection	Pregnancy	Post-treatment	Normal values
Ferritin levels	164µg/L	48µg/L	8µg/L	31µg/L	12-300µg/L
Transferrin levels	74%	48%	9%	60%	25-50%
Hb levels	124g/L	122g/L	102g/L	133g/L	115-165g/L

Her iron levels remained depleted after pregnancy with symptoms of fatigue and exhaustion which were affecting her quality of life. However, her Hb and iron levels improved to normal after being commenced on a short course of iron supplementation after which her bloods were subjected to ongoing close surveillance in the community and by the gastroenterology team as part of a shared care plan.

**Figure 1:**
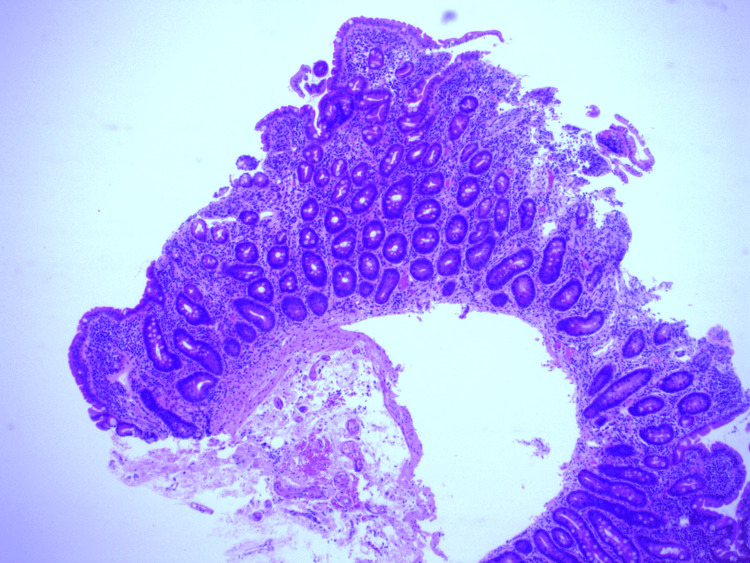
Duodenum histology image showing villous atrophy in keeping with CD. CD: celiac disease

**Figure 2:**
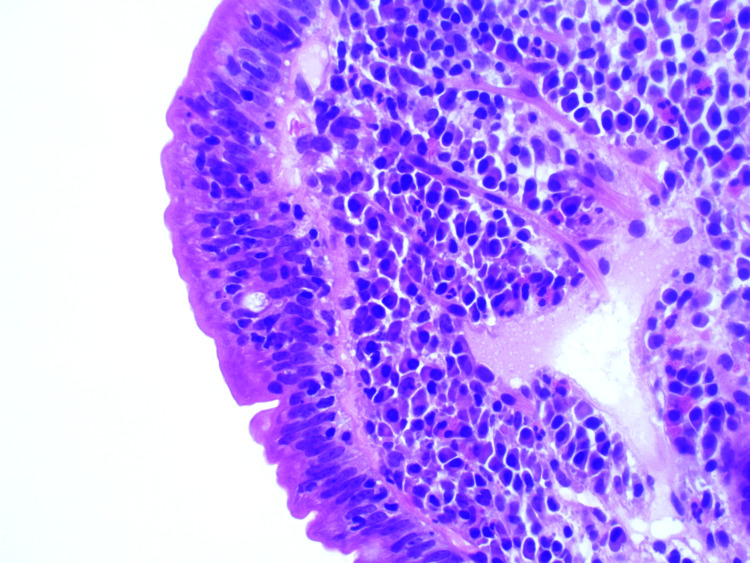
Duodenal biopsy's histology image showing increased intraepithelial lymphocytes in keeping with CD. CD: celiac disease

The patient initially presented in 2013 with stomach pain, diarrhea, and fatigue. She denied any family history of CD though one of her parents was given a diagnosis of irritable bowel syndrome. She had one previous pregnancy with no complications. Her blood tests showed a low iron level with a ferritin level of 52µg/L (normal range: 10-290µg/L), slightly elevated transferrin saturation of 53% (normal range: 15-50%), and raised immunoglobulin A (IgA) TTG antibodies of >80U (normal range: 0-6.9U). Other investigations were normal including FBC, thyroid function test, renal function test, liver function test, HBA1C, serum ferritin, and vitamin B12. She was referred for endoscopy, but due to the severity of her symptoms, she restarted a gluten-free diet herself, and therefore, gastroscopy was normal macroscopically. However, the histopathology report of her duodenal biopsy revealed variable villous atrophy and increased intraepithelial lymphocytes (Figure 1 and Figure 2). One small fragment showed almost complete villous atrophy, while others showed partial villous atrophy.

These findings combined with the positive serology were quite diagnostic for CD. The patient reported an improvement in symptoms as soon as she was established on the gluten-free diet, and her CD remained in remission for eight years as evidenced by symptom control, ferritin repletion, and normal serology results in 2021.

In 2021, she was offered to be screened for HH after her sister had been diagnosed with the condition. Her genes came back as homozygous for the C282Y variant. On further exploration of her symptoms, she reported arthralgia in the small joints of her hands along with fatigue. She otherwise led a healthy lifestyle and did not smoke or drink alcohol. Her highest recorded ferritin level was 164µg/L and her transferrin saturation was 74% in 2021 (Table 1).

**Figure 3:**
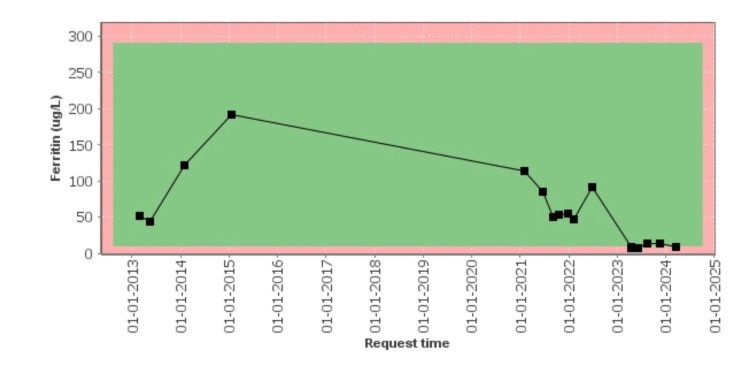
Trend of ferritin levels.

Other blood investigations including FBC, HBA1C, and ultrasound (US) of the liver were unremarkable. She was reviewed in the clinic and commenced on venesection. Her symptoms of arthralgia and fatigue improved upon commencing on venesection in September 2021. She had 12 weekly sessions of venesections, and her ferritin levels and transferrin saturations responded to be within the target range of <50µg/L and <50%, respectively (Table 1). She subsequently became pregnant with her second child which precipitated her iron depletion resulting in iron deficiency anemia in her late second to third trimester. Her ferritin level dropped significantly to 8µg/L and Hb to 102g/L during this time (Table 1, Figure 3).

Her gastroenterologist advised to stop venesection and recommended for frequent blood monitoring during pregnancy with a view to avoid iron deficiency and potentially start iron replacement if needed. She successfully delivered her baby with an elective caesarian section. Her Hb level improved to 122g/dL without any intervention, but her ferritin level remained low for nearly a year in the postpartum period; therefore, she took iron supplements for a short duration of time. Due to severe fatigue and exhaustion, she was started on iron tablets on alternate days for two weeks during March 2024, and her ferritin levels improved to 31µg/L (Table 1). Her blood tests were monitored in the community with an aim to keep her Hb in normal range and for her ferritin to be between 30µg/L and 50µg/L. She was also monitored and managed by the gastroenterology team and listed for a patient-initiated pathway with set targets of ferritin levels between 30µg/L and 50µg/L.

## Discussion

Our case highlights the rare co-existence of HH and CD and the challenge in diagnosing and managing them due to their opposing interactions on iron storage in the body. Similarly, to our knowledge, this is the only case in recorded literature that explores the impact of pregnancy for patients with these dual conditions and also highlights the effect of postnatal period on iron homeostasis in the body. It is worthwhile noting that this patient did not have any issues with iron deficiency anemia during her first pregnancy when she had not been diagnosed with HH which could potentially emphasize that the iron overload state from her undiagnosed HH may be playing a protective role against the development of iron deficiency anemia. This case also illustrated the importance and requirement for closely monitoring these patients to prevent both iron deficiency anemia and iron overload as well as signifying the shared care through close communication between gastroenterology and obstetric teams and the community during pregnancy in order to achieve ideal outcomes for patients. Previous case reports by Heneghan et al. and Singhal et al. on this highly unusual phenomenon report similar findings to our case in that the manifestation of hemochromatosis is delayed by the co-existence of CD [9,10]. This may be due to the decreased iron absorption from the downregulation of DMT1 [11]. Even when CD is well controlled on diet, iron deficiency may still impact patients which may be due to non-healing at an ultrastructural level even as the villi may have appeared to have healed macroscopically [12]. In contrast, it is also reported that HH causes an upregulation of the DMT1 protein which increases iron absorption. Therefore, it was only when the CD was controlled that the clinical manifestations of HH started to present and may in fact have been masked during the first pregnancy. Some authors have in fact quoted a relationship between people with CD and mutations of the HFE gene as described in these two examples: Ludvigsson et al. explored the relationship between CD and HH and through their case-control study reported that there was an increased propensity for patients with CD to have HH. Butterworth et al. reported the same and hypothesized that this may be due to the survival advantage conferred by HH on patients with CD [13,14].

## Conclusions

Our case highlights the challenges in diagnosing and managing patients with concurrent CC and HH due to their opposing effects on iron absorption and metabolism and shines a light on the challenges and care needed to manage these dual conditions in pregnancy due to the overall important role of iron inside and outside of this period. It can be deduced concretely that the management of iron levels in such patients required a holistic and multi-faceted approach by healthcare teams. It emphasizes the importance of managing these patients in a multidisciplinary approach by involving a general physician, gynecologist, gastroenterologist, and hematologist to provide adequate surveillance and intervention if need be and achieve the correct balance between iron storage of the body in order to prevent potential associated hepatic and extrahepatic complications of iron overload and depletion secondary to these two intricate conditions. Furthermore, it also demonstrates that uncontrolled CD and pregnancy may delay and mask the presentation of hemochromatosis or vice versa.
